# Unveiling the importance of the interface in nanocomposite cathodes for proton‐conducting solid oxide fuel cells

**DOI:** 10.1002/EXP.20230082

**Published:** 2024-02-01

**Authors:** Yanru Yin, Yifan Wang, Nan Yang, Lei Bi

**Affiliations:** ^1^ School of Resources Environment and Safety Engineering University of South China Hengyang China; ^2^ Electrochemical thin film group, School of Physical Science and Technology ShanghaiTech University Shanghai P. R. China; ^3^ Division of Physical Sciences and Engineering King Abdullah University of Science and Technology (KAUST) Thuwal Saudi Arabia

**Keywords:** interface, proton conductor, pulsed laser deposition, solid oxide fuel cells

## Abstract

Designing a high‐performance cathode is essential for the development of proton‐conducting solid oxide fuel cells (H‐SOFCs), and nanocomposite cathodes have proven to be an effective means of achieving this. However, the mechanism behind the nanocomposite cathodes' remarkable performance remains unknown. Doping the Co element into BaZrO_3_ can result in the development of BaCoO_3_ and BaZr_0.7_Co_0.3_O_3_ nanocomposites when the doping concentration exceeds 30%, according to the present study. The construction of the BaCoO_3_/BaZr_0.7_Co_0.3_O_3_ interface is essential for the enhancement of the cathode catalytic activity, as demonstrated by thin‐film studies using pulsed laser deposition to simulate the interface of the BCO and BZCO individual particles and first‐principles calculations to predict the oxygen reduction reaction steps. Eventually, the H‐SOFC with a BaZr_0.4_Co_0.6_O_3_ cathode produces a record‐breaking power density of 2253 mW cm^−2^ at 700°C.

## Graphical abstract

The interface of nanocomposite cathode plays a critical role in the performance of proton‐conducting solid oxide fuel cells (H‐SOFCs), which has been demonstrated by atomic level studies with the first‐principles calculations, nanoscale studies by using the pulsed laser deposition method and the prototype fuel cell studies. The resultant BaZr_0.4_Co_0.6_O_3_ cathode leads to a record‐breaking power density for H‐SOFCs.

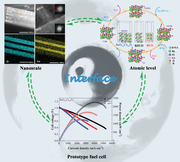

## 1 INTRODUCTION

The world's present energy and environmental concerns necessitate the development of renewable and sustainable technology.^[^ ^1^ ^]^ In recent decades, fuel cell technology, which can directly transform chemical energy into electricity, has garnered substantial attention.^[^ ^2^ ^]^ Among the several forms of fuel cells, solid oxide fuel cells (SOFCs) with their solid structures have become a popular study area.^[^ ^3^ ^]^ Traditional SOFCs must operate at high temperatures, resulting in a decreased fuel cell lifetime, interfacial diffusions, and challenges in selecting appropriate materials.^[^ ^4^ ^]^ Proton‐conducting SOFCs (H‐SOFCs) offer a possible approach for lowering the operating temperature of SOFCs.^[^ ^5^ ^]^ H‐SOFCs are proton‐conducting electrolyte‐based SOFCs.^[^ ^6^ ^]^ Due to the high electrolyte protonic conductivity at intermediate temperatures, the working temperatures of H‐SOFCs are lower compared to those of traditional SOFCs.^[^ ^7^ ^]^ However, the decreased operating temperature also reduces the cathode kinetics, resulting in the need for high‐performance cathodes.^[^ ^8^ ^]^

In general, the design of high‐performance cathodes focuses primarily on the usage of composite^[^ ^9^ ^]^ or triple‐conduction cathodes.^[^ ^10^ ^]^ The composite cathode, which consists of a proton‐conducting phase and an electron‐conducting phase, is the most common cathode configuration for H‐SOFCs. The composite cathode design can extend the triple‐phase boundaries (TPBs) to the connections between the two phases, thereby expanding the active reaction area and enhancing cathode performance. In contrast, triple‐conduction cathodes, in which protons, oxygen‐ions, and electrons can migrate, have been established in recent years.^[^ ^11^ ^]^ In theory, the TPBs are extended to the whole surface of the triple‐conduction cathode, as opposed to the standard composite cathodes, which only have two‐phase connections. Consequently, cathode performance can be enhanced, along with fuel cell performance.^[^ ^12^ ^]^

Recently, nanocomposite cathodes, a new cathode design with promising performance for H‐SOFCs, were proposed.^[^ ^13^ ^]^ In contrast to conventional composite cathodes, the nanocomposite cathode is comprised of two mixed conducting phases generated from a single‐pot synthesis, providing a novel cathode design for H‐SOFCs. Despite the fact that great performance has been attained using nanocomposite cathodes, the performance increase mechanism has not yet been identified. The high performance may be attributed to the enhanced oxygen reduction reaction (ORR) activity; however, the origin of the high ORR activity is unclear. The TPB region of nanocomposite cathodes is not as extensive as that of triple‐conduction cathodes that expand the TPBs throughout the entire cathode surface. However, the performance of the reported nanocomposite cathodes is comparable to or even greater than that of some triple‐conduction cathodes, indicating that factors other than the cathode material's properties have a major impact on the cathode's overall performance.

In this study, a high‐performance nanocomposite cathode for H‐SOFCs is fabricated by modifying the conventional BaZrO_3_ proton conductor with Co cations. Encouragingly high fuel cell performance has been attained, and it has been determined that the interface is the origin of the higher performance in the nanocomposite cathode. The significance of the interface in the nanocomposite cathode has been proven at the atomic level with density functional theory (DFT) calculations, at the nanoscale with thin‐film investigations utilizing the pulsed laser deposition (PLD) technique, and with prototype fuel cell testing.

## 2 RESULTS AND DISCUSSIONS

**FIGURE 1:**
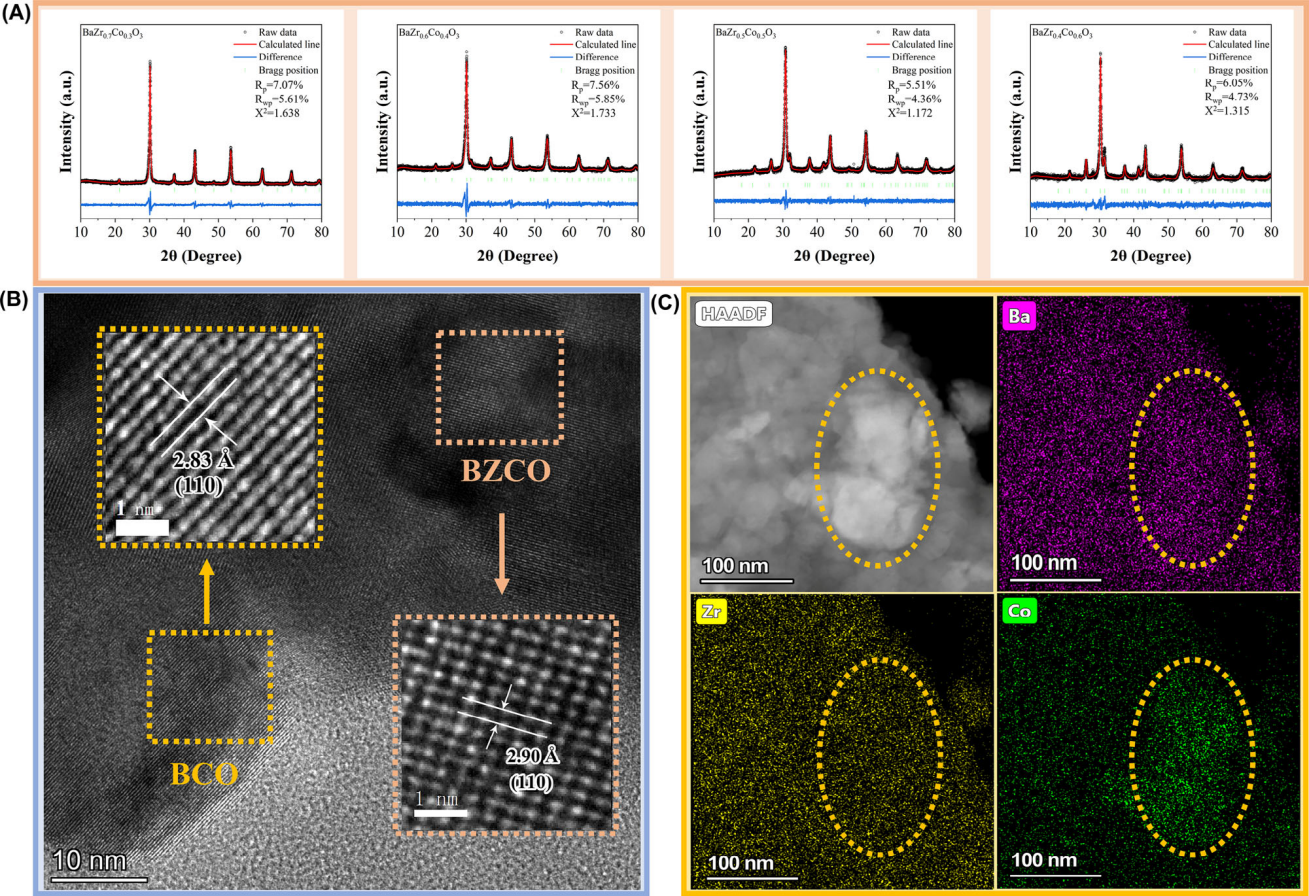
(A) Rietveld Refinement results for BaZr_0.7_Co_0.3_O_3_ (BZCO), BaZr_0.6_Co_0.4_O_3_, BaZr_0.5_Co_0.5_O_3_ and BaZr_0.4_Co_0.6_O_3_; (B) HRTEM for BaZr_0.4_Co_0.6_O_3_; (C) HAADF image and corresponding elemental mapping results for BaZr_0.4_Co_0.6_O_3_.

Figure S1, Supporting Information depicts X‐ray diffraction (XRD) patterns of the as‐prepared BaZr_1−_ *_x_* Co*_x_* O_3_ (*x* = 0.3, 0.4, 0.5, and 0.6) oxides, which were synthesized using the usual sol–gel technique. One can observe that a pure phase can be formed in the material with a Co‐doping concentration of up to 30%, which translates to a pure phase Co‐doped BaZrO_3_ concentration of up to *x* = 0.3. Once the Co doping approaches 40%, a secondary phase corresponding to BaCoO_3_ (BCO) becomes apparent.^[^ ^14^ ^]^ The amount of BCO grows as the concentration of Co rises. Figure 1A shows the Rietveld Refinement results for BaZr_0.7_Co_0.3_O_3_ (BZCO), BaZr_0.6_Co_0.4_O_3_, BaZr_0.5_Co_0.5_O_3_ and BaZr_0.4_Co_0.6_O_3_ to further understand the phase separation. A pure cubic phase is detected for BZCO with the space group of *Pm‐3m* and the lattice parameters of *a* = *b* = *c* = 4.092 Å; while the refinement results of BaZr_0.6_Co_0.4_O_3_, BaZr_0.5_Co_0.5_O_3_ and BaZr_0.4_Co_0.6_O_3_ are the compositions of cubic BZCO, which is the dominant phase, and hexagonal phase (space group of *P63/mmc*), BaCoO_3_ (BCO), with the lattice parameter of *a* = *b* = 5.68 Å, *c* = 4.76 Å. The reliable Rietveld refinement results can be confirmed by the reasonable value of weighted residuals (*R*_wp_), the least squares residuals (*R*_p_), and the goodness of fitted R‐factors (*χ* ^2^ = 1.638, 1.733, 1.172, 1.315 for BZCO, BaZr_0.6_Co_0.4_O_3_, BaZr_0.5_Co_0.5_O_3_, and BaZr_0.4_Co_0.6_O_3_, respectively). BaZr_0.4_Co_0.6_O_3,_ with the highest amount of BCO among all the samples, was further studied to understand the existence of two phases, as shown in the high‐resolution transmission electron microscopy (HR‐TEM) images of Figure 1B. Fast Fourier transform (FFT) and inversed FFT technique was applied by using the software DigitalMicrgraph to clarify the d‐spacing of 2.83 Å for the BCO phase and 2.90 Å for BZCO phase corresponding to the crystal plane (110), respectively. This result confirms well with the results of Rietveld refinement for BaZr_0.4_Co_0.6_O_3_. The associated high‐angle annular dark field (HAADF) image and energy dispersive spectroscopy (EDS) mapping result shown in Figure 1C is another strong demonstration of the existence of phase separation, the elemental accumulation can be observed, which is attributed to the phase of BCO. These results further confirm the nominal BaZr_0.4_Co_0.6_O_3_ material is the nanocomposite of BZCO+BCO.

The design of the BZCO+BCO composite enhances the oxygen vacancy content at the surface. Figure S2, Supporting Information illustrates the XPS O 1s spectrum of BCO, BZCO, and BaZr_0.4_Co_0.6_O_3_ material. It has been reported that the ratio between the adsorbed oxygen and the lattice oxygen reflects the surface oxygen vacancy (Vo) content,^[^ ^15^ ^]^ and the ratios for BCO, BZCO, and BaZr_0.4_Co_0.6_O_3_ are 1.33, 0.9, and 2.06, respectively, indicating that the formation of BZCO+BCO in BaZr_0.4_Co_0.6_O_3_ increases the Vo content. As Vo has a significant impact on the ORR activity and oxygen mobility,^[^ ^16^ ^]^ BaZr_0.4_Co_0.6_O_3_ should have a higher ORR activity than BCO and BZCO. The findings of electrical conductivity relaxation (ECR) measurements for BCO, BZCO, and BaZr_0.4_Co_0.6_O_3_ are depicted in Figure S3, Supporting Information. By changing the environment from air to 50% O_2_, the conductivity of the samples changes, and the relaxation time indicates the oxygen diffusion capacity and surface oxygen exchange rate. One may observe that the BCO and BZCO relaxation times are several thousand and tens of thousands of seconds, respectively. In comparison, BaZr_0.4_Co_0.6_O_3_ has a relaxation time of only a few hundred seconds. The oxygen diffusion coefficient (D*) is calculated to be 7.78×10^−6^, 2.29×10^−6^ and 9.66×10^−5^ cm^2^ s^−1^, and the oxygen surface exchange coefficient (k*) is 9.81×10^−5^, 2.88×10^−5^ and 1.17×10^−3^ cm s^−1^ for BCO, BZCO and BaZr_0.4_Co_0.6_O_3_, respectively. The D* and k* values of BaZr_0.4_Co_0.6_O_3_, which is the BZCO+BCO nanocomposites, are at least one order of magnitude larger than those for BCO or BZCO, suggesting the oxygen diffusion and exchange ability is greatly improved with the formation of BCO/BZCO interfaces.

**FIGURE 2:**
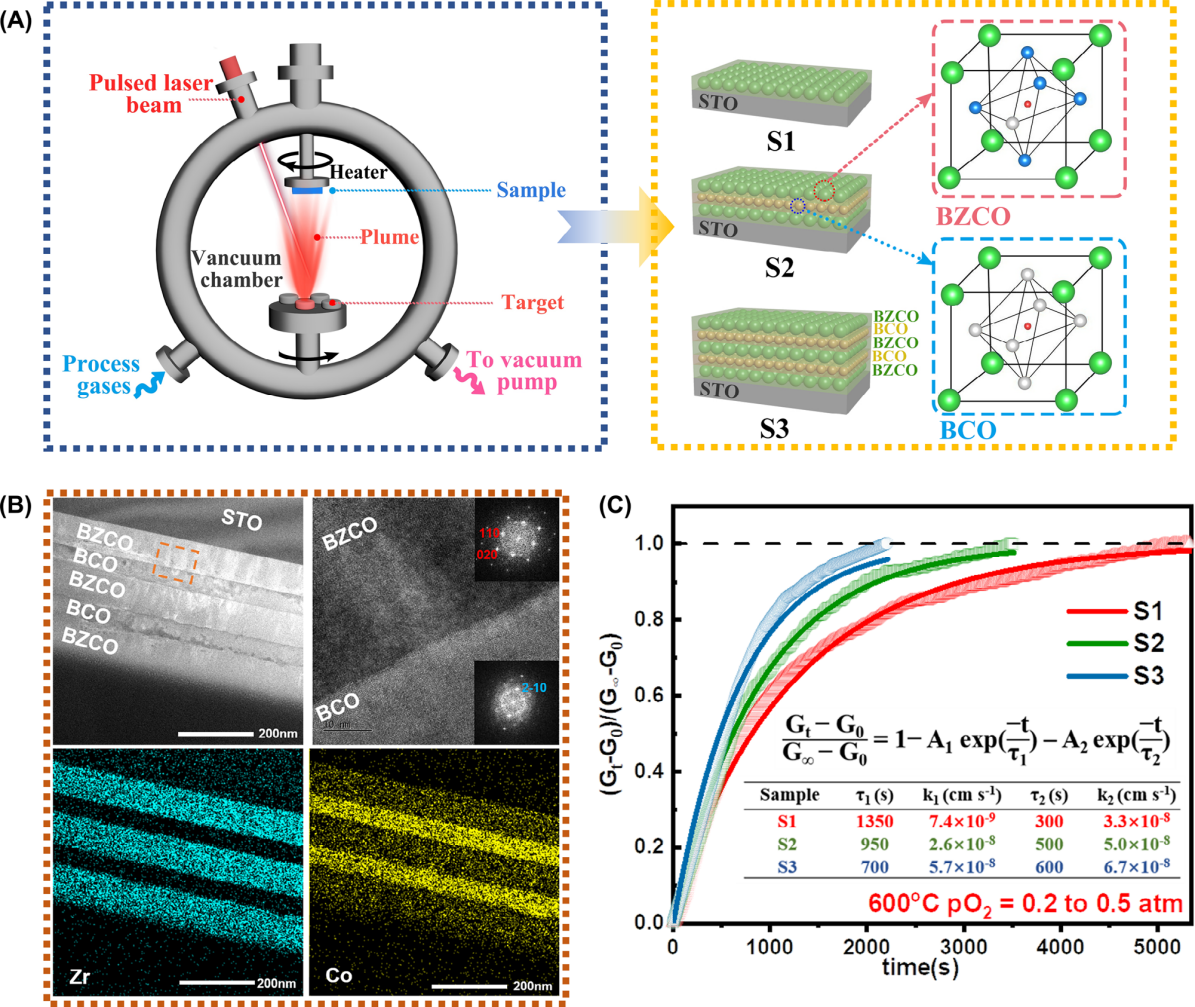
(A) Schematic diagrams of sample fabrication of BZCO and BCO multilayers by PLD. (B) Cross‐sectional bright field TEM images of five‐layer BZCO/BCO film and the corresponding EDS mappings of Zr and Co. In addition, a higher magnification image of selected area demonstrates the interface between BZCO and BCO with the corresponding SAED patterns. (C) ECR measurements of BZCO single layer and BZCO and BCO multilayers.

Notably, the aforementioned experimental results are still derived from the bulk materials as a whole, and it would be interesting to compare the characteristics of single BCO or BZCO particles with BCO/BZCO composite particles to confirm the impact of the interface. The pulsed laser disposition (PLD) is a powerful tool for studying the reaction mechanism by constructing nanoscale thin films to simulate the reaction at nanometric particles, despite the technical difficulty of measuring individual nanoscale particles.^[^ ^17^ ^]^ In order to elucidate the significance of the interface in the cathode reaction, several thin films were manufactured. In specific, the single‐layer (BZCO, S1), three‐layer (BZCO/BCO/BZCO, S2), and five‐layer (BZCO/BCO/BZCO/BCO/BZCO, S3) samples were grown by PLD on (100)‐oriented single crystalline SrTiO_3_ (STO) substrates, as schemed in Figure 2A. The crystallographic quality of thin films is confirmed by using XRD measurements. As shown in Figure S4, Supporting Information, only (00l) diffraction peaks from BZCO and BCO can be observed apart from the STO substrate, indicating a strong [001] preferential orientation for thin films with good crystallographic quality. Figure 2B displays the cross‐sectional TEM images and the related EDS mappings for the S3 sample. Bright‐field HRTEM images reveal the formation of a multilayer structure of BZCO/BCO with well‐defined interfaces between layers. The BZCO and BCO layers have a thickness of 85 and 47 nm, respectively. EDS mappings demonstrate homogeneous distributions of Zr and Co in BZCO and BCO layers, with no detectable element interdiffusion at the interfaces, suggesting that the sample structures are of high quality. The local crystal structure information was obtained by performing the Fourier transmission (FT) analysis of the orange region indicated in TEM Figure 2B. The BZCO grows on STO with the crystallographic relationship as (001) BZCO|| (001) STO. However, the more diffused FT pattern indicates that the BCO layer grows more disordered than the BZCO layer and the (2‐10) orientation can be obtained.

The impact of the interface on the surface oxygen exchange process was investigated by ECR measurements. The relaxation curve of S1, S2, and S3 samples at 600°C with a given change in oxygen partial pressure can be found in Figure 2C. The relaxation profiles of thin films with a thickness inferior to the critical thickness reveal more information on the surface exchange reaction process rather than the bulk diffusion of oxygen.^[^ ^18^ ^]^ The double‐exponential function was applied to fit the relaxation curves, which is commonly used in the process of samples with microstructural inhomogeneity.^[^ ^19^ ^]^ The relaxation time (*τ*_1_, *τ*_2_) and the calculated surface oxygen exchange coefficients (*k*_1_, *k*_2_), representing the slow and fast kinetic processes, are shown in Figure 2C. The k_1_ value shows clear dependence on the number of interfaces. Specifically, the k_1_ increases from 7.4×10^−9^ cm s^−1^ of S1 to 2.6×10^−8^ cm s^−1^ S2 and eventually to 5.7×10^−8^ cm s^−1^ of S3. This result indicates that the interface between BZCO and BCO plays a crucial role in promoting surface oxygen exchange kinetics. In addition, the interface also influences the conductivities of the thin films, as shown in Figure S5, Supporting Information. One can see that the conductivity gradually increases with the number of interfaces, indicating the critical role of the interface in boosting the performance of nanocomposite cathodes.

**FIGURE 3:**
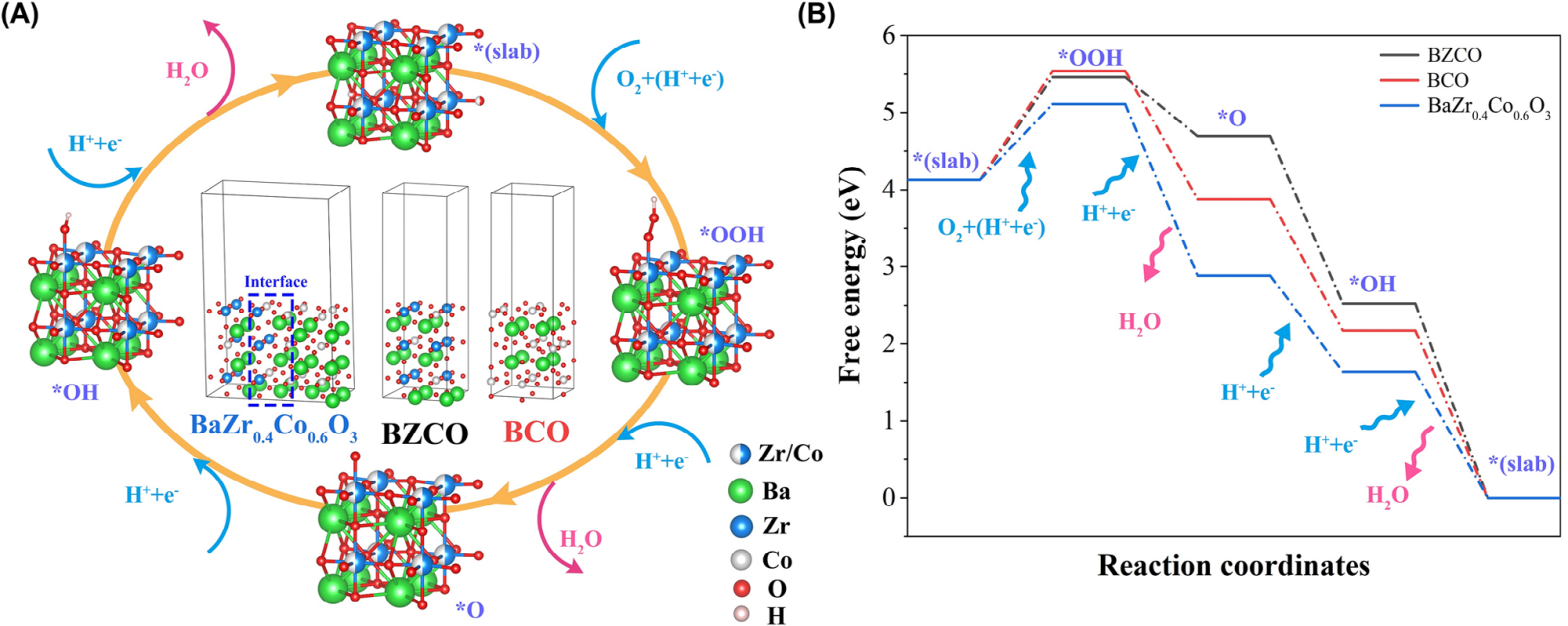
(A) Scheme of the ORR procedure at BCO, BZCO, and BCO/BZCO interface; (B) Free energy of the ORR procedure at BCO, BZCO, and BCO/BZCO interface.

Using DFT calculations, the role of the BCO/BZCO interface for the ORR activity is further elaborated at the atomic level. In the H‐SOFCs' cathode catalytic process, the delivered protons combined with O_2_ to generate H_2_O. The chemical reaction equation can be written as: $4H^{+}+O2\to 2H2O$. As illustrated in Figure 3A, the ORR for the H‐SOFC cathodes involves the formation of *OOH, *O, and *OH intermediates, as well as the release of H_2_O molecules.^[^ ^20^ ^]^ Figure 3B depicts the results of calculating the free energy for these reaction steps at 600°C on BCO, BZCO, and BCO/BZCO interface in order to examine the impact of the interface on the ORR activity. The formation of *OOH is the energy‐determining process in all these three models. However, the energy barrier for the formation of *OOH at the BCO/BZCO interface is much lower than that at the BCO and BZCO, resulting in a lower energy barrier for ORR at the BCO/BZCO interface. Therefore, the construction of BCO/BZCO interfaces in a material can promote the cathode reaction.

**FIGURE 4:**
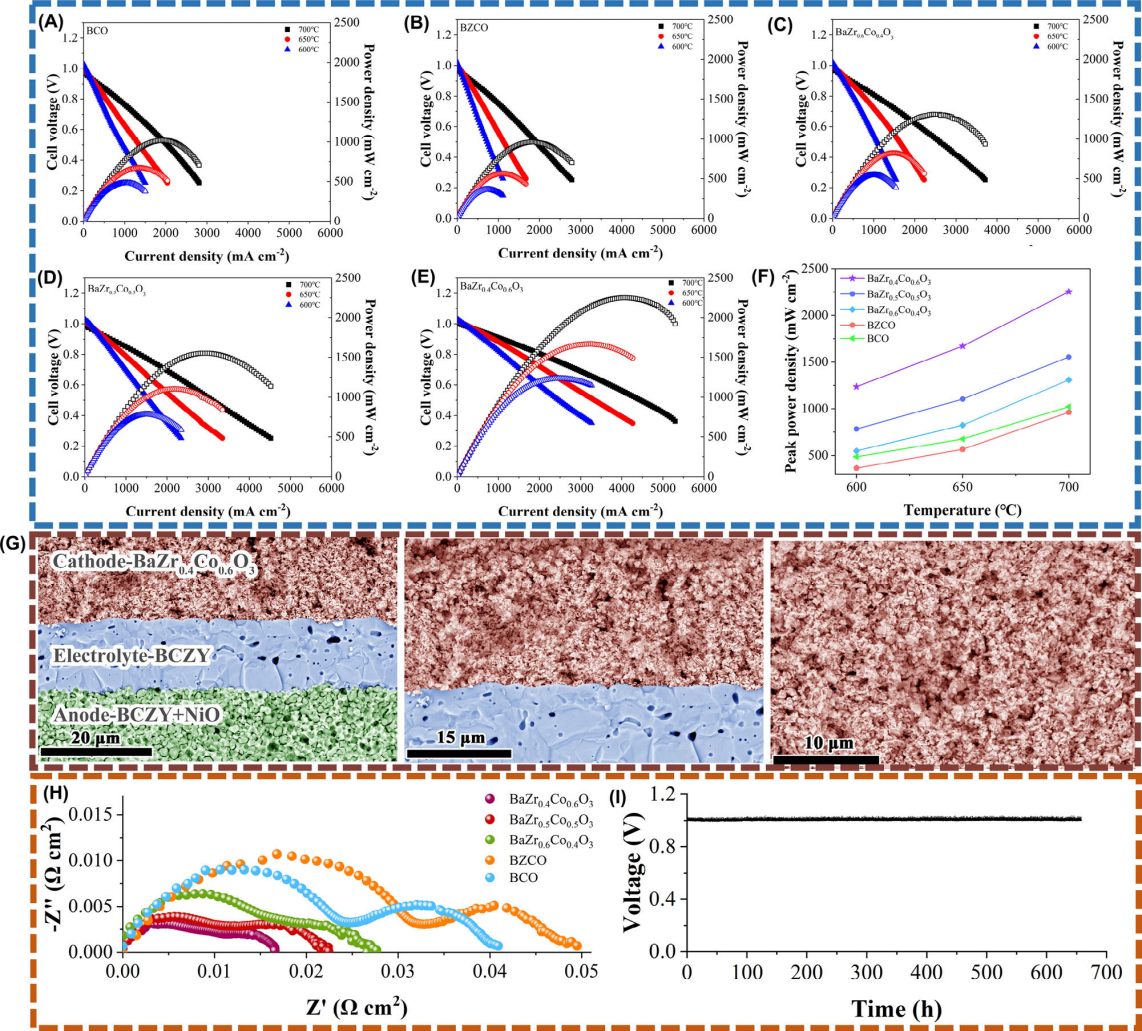
Fuel cell performance of an H‐SOFC using (A) BCO, (B) BZCO, (C) BaZr_0.6_Co_0.4_O_3_, (D) BaZr_0.5_Co_0.5_O_3,_ and (E) BaZr_0.4_Co_0.6_O_3_ cathodes, respectively; (F) comparison of peak power densities of these different cells; (G) cross‐sectional view of the BaZr_0.4_Co_0.6_O_3_ cell after testing; (H) comparison of Rp for the cells using BCO, BZCO, BaZr_0.6_Co_0.4_O_3_, BaZr_0.5_Co_0.5_O_3_, and BaZr_0.4_Co_0.6_O_3_ cathodes; (I) long‐term stability for the BaZr_0.4_Co_0.6_O_3_ cell under the working condition at 600°C with an applied current density of 200 mA cm^−2^.

Figures 4A–E depict the performance of H‐SOFCs with the respective cathodes BCO, BZCO, BaZr_0.6_Co_0.4_O_3_, BaZr_0.5_Co_0.5_O_3_, and BaZr_0.4_Co_0.6_O_3_. Clearly, the performance of the fuel cells with the BaZr_0.6_Co_0.4_O_3_, BaZr_0.5_Co_0.5_O_3_, and BaZr_0.4_Co_0.6_O_3_ cathodes is superior to that of the cells with the single‐phase BCO or BZCO cathode. At 700°C, the peak power density (PPD) for BCO and BZCO cells is 1021 and 962 mW cm^−2^, respectively. Under the same testing conditions, the PPD for the BaZr_0.6_Co_0.4_O_3_, BaZr_0.5_Co_0.5_O_3_, and BaZr_0.4_Co_0.6_O_3_ cells is 1309, 1554, and 2253 mW cm^−2^, respectively. The performance of the BaZr_0.6_Co_0.4_O_3_, BaZr_0.5_Co_0.5_O_3_, and BaZr_0.4_Co_0.6_O_3_ cells is higher than that for BCO and BZCO cells at the entire testing temperature range as shown in Figure 4F. As the BCO, BZCO, BaZr_0.6_Co_0.4_O_3_, BaZr_0.5_Co_0.5_O_3_, and BaZr_0.4_Co_0.6_O_3_ cells show similar fuel cell morphologies, as indicated in Figure 4G and Figure S6, Supporting Information, the different cell performance should mainly come from the different cathode material used. As BaZr_0.6_Co_0.4_O_3_, BaZr_0.5_Co_0.5_O_3_, and BaZr_0.4_Co_0.6_O_3_ are composites of BCO+BZCO, the better fuel cell performance suggests that nanocomposition design is essential for cell performance. It is also observed that the performance of the fuel cell improves from BaZr_0.6_Co_0.4_O_3_ to BaZr_0.5_Co_0.5_O_3_ and then to BaZr_0.4_Co_0.6_O_3_.

This result confirms that the greater the number of BCO/BZCO interfaces, the higher the performance of the fuel cell, and that the formation of the BCO/BZCO interface is crucial for the cathode reactions and thus improves the cell performance. The mechanism for this improvement has been elaborated by experimental studies and DFT calculations, as mentioned above. To the best of our knowledge, the cell using the BaZr_0.4_Co_0.6_O_3_ cathode presents the power density at the high level for H‐SOFCs, as indicated in Table 1 that summaries recently reported high‐performing H‐SOFCs in the literature,^[^ ^8^, ^12^, ^13^, ^21^ ^]^ suggesting BaZr_0.4_Co_0.6_O_3_ is an efficient cathode for H‐SOFCs. To exclude the influence of possible interfacial reaction between BaZr_0.4_Co_0.6_O_3_ and BCZY electrolyte, the chemical compatibility between BaZr_0.4_Co_0.6_O_3_ and BCZY is examined, and the result is shown in Figure S7, Supporting Information. No reaction for the BaZr_0.4_Co_0.6_O_3_+BCZY composite powder can be detected after co‐firing, suggesting good chemical compatibility between BaZr_0.4_Co_0.6_O_3_ and BCZY.

**TABLE 1:** Comparison of fuel cell performance between the cell reported in this study with other recently reported high‐performing H‐SOFCs. BCZYYb: BaCe_0.7_Zr_0.1_Y_0.1_Yb_0.1_O_3_; BCMF: BaCe_0.8_Sm_0.2_F_0.1_O_2.85_; BCZY442: BaCe_0.4_Zr_0.4_Y_0.2_O_3_; BCZYYb4411: BaCe_0.4_Zr_0.4_Y_0.1_Yb_0.1_O_3_; BCZY: BaCe_0.7_Zr_0.1_Y_0.2_O_3_.

Cathode composition	Anode and electrolyte	PPD (mW cm^−2^)	Year^[Ref.]^
PrNi_0.5_Mn_0.5_O_3_+PrO_2_ nanocomposite	Ni+BCZYYb/ BCZYYb	650 (700°C)	2018^[^ ^21a^]
Ba_0.5_Sr_0.5_Co_0.8_Fe_0.2_O_2.9−_ *_δ_* F_0.1_	Ni+BCMF /BCMF	480 (700°C)	2018^[^ ^21b^ ^]^
BaCo_0.9_(Ce_0.8_Y_0.2_)_0.1_O_3_+BaCoO_3_ nanocomposite	Ni+BCZYYb/BCZYYb	985 (650°C)	2019^[^ ^13a^ ^]^
	Ni+BCZY442/BCZY442	464 (650°C)	
BaCe_0.4_Fe_0.4_Co_0.2_O_3_ nanocomposite	Ni+BCZYYb/BCZYYb	335 (700°C)	2020^[^ ^11d^ ^]^
PrNi_0.5_Co_0.5_O_3−_ *_δ_*	Ni+BCZYYb4411/BCZYYb4411	528 (650°C)	2020^[^ ^12^ ^]^
Sm_0.5_Sr_0.5_CoO_3−_ *_δ_* +SmBaCo_2_O_5+_ *_x_* nanocomposite	Ni+BCZYYb/BCZYYb	1570 (750°C)	2020^[^ ^21c^ ^]^
La_0.5_Sr_1.5_MnO_4_+La_0.5_Sr_0.5_MnO_3_ nanocomposite	Ni+BCZY/BCZY	936 (700°C)	2020^[^ ^21d^ ^]^
Pr_2_BaNiMnO_7_	Ni+BCZYYb/BCZYYb	1070 (700°C)	2020^[^ ^21e^ ^]^
La_0.25_Sr_2.75_FeNiO_7−_ *_δ_*	Ni+BCZY/BCZY	426 (650°C)	2021^[^ ^21f^ ^]^
(La_0.6_Sr_0.4_)_0.95_Co_0.2_Fe_0.8_O_3_+Pr_1−_ *_x_* Ba*_x_* CoO_3_+BaCoO_3_ nanocomposite	Ni+BCZYYb/BCZYYb	1040 (600°C)	2022^[^ ^8b^ ^]^
La_0.5_Sr_0.5_Mn_0.875_Sc_0.125_O_3_	Ni+BCZY/BCZY	1221 (700°C)	2022^[^ ^21g^ ^]^
Sr_2_Fe_1.5_Mo_0.25_Sc_0.25_O_6−_ *_δ_*	Ni+BCZY/BCZY	1258 (700°C)	2022^[^ ^21h^ ^]^
La_0.5_Sr_0.5_Fe_0.9_P_0.1_O_3−_ *_δ_*	Ni+BCZY/BCZY	1322 (700°C)	2022^[^ ^21i^ ^]^
Ba_0.95_Fe_0.7_Co_0.2_Sc_0.1_O_3_	Ni+BCZYYb/BCZYYb	522 (600°C)	2022^[^ ^21j^ ^]^
BaCe_0.36_Fe_0.64_O_3_ nanocomposite	Ni+BCZY/BCZY	1525 (700°C)	2022^[^ ^21k^ ^]^
Ba_0.875_Fe_0.875_Zr_0.125_O_3_	Ni+BCZYYb/BCZYYb	2040 (700°C)	2022^[^ ^21l^ ^]^
BaCe_0.16_Y_0.04_Fe_0.8_O_3_ nanocomposite	Ni+BCZYYb/BCZYYb	829 (650°C)	2022^[^ ^13b^ ^]^
La_0.5_Ba_0.5_MnO_3_ nanocomposite	Ni+BCZY/BCZY	1504 (700°C)	2023^[^ ^21n^ ^]^
Pr_0.5_Sr_0.5_MnO_3_+PrO_2_ nanocomposite	Ni+BCZY/BCZY	1446 (700°C)	2023^[^ ^21o^ ^]^
BaZr_0.4_Co_0.6_O_3_ nanocomposite	Ni+BCZY/BCZY	2253 (700°C)	This study
		1671 (650°C)	
		1236 (600°C)	

Although the BaZr_0.4_Co_0.6_O_3_ cell and other cells present similar cell morphologies, the polarization resistance of the cell (*R*_p_) decreases from BaZr_0.6_Co_0.4_O_3_ to BaZr_0.5_Co_0.5_O_3_ to BaZr_0.4_Co_0.6_O_3_, and these *R*_p_ values are lower than those for BCO or BZCO cathodes, as shown in Figure 4H, suggesting the formation of BCO/BZCO interface can accelerate the cathode reaction and the more interface, the faster cathode kinetics. The area‐specific resistance (ASR) tests based on BCZY‐supported symmetrical cells show similar results, as presented in Figure S8, Supporting Information. The BaZr_0.4_Co_0.6_O_3_ cathode shows the lowest ASR values among all the testing cathodes. The *R*_p_ for the cell using BaZr_0.4_Co_0.6_O_3_ cathode is only 0.0165 Ω cm^2^. This *R*_p_ is the smallest reported for cathodes of H‐SOFCs. In addition to the improved cell output, the nanocomposite cathode enhances cell stability in the fuel cell operating conditions. Figure S9, Supporting Information shows the stability test of the cells using BCO, BZCO, and BaZr_0.4_Co_0.6_O_3_ cathodes. Apparent cell degradation can be observed for the BCO and BZCO cells, whereas the BaZr_0.4_Co_0.6_O_3_ cell remains stable. The degradation rate is around 7.5% and 7.9% for the BCO and BZCO cells, respectively, after the short‐term operation. In contrast, the voltage remains at 1.00 V for the BaZr_0.4_Co_0.6_O_3_ cell without evident drop. It has been reported that the grain growth of nanocomposites can be restricted compared with individual components.^[^ ^4c^ ^]^ In this case, the long‐term stability of the cathode and the fuel cell can be improved. The excellent stability of the BaZr_0.4_Co_0.6_O_3_ cell is further demonstrated with a stable operation under the fuel cell working condition for 650 h, as shown in Figure 4I. No evident degradation of the BaZr_0.4_Co_0.6_O_3_ cell can be observed. The ultra‐high fuel cell performance and the excellent stability of the BaZr_0.4_Co_0.6_O_3_ cell indicate that the BaZr_0.4_Co_0.6_O_3_, of which the high catalytic activity originated from the BCO/BZCO interface, is a highly‐efficient and robust cathode for H‐SOFCs.

If one further increases the Co‐content in BaZr_1−_ *_x_* Co*_x_* O_3_ cathodes, the fuel cell performance does not continue to increase, as shown in Figure S10, Supporting Information. When *x* = 0.7, the PPD of the cell using the BaZr_0.3_Co_0.7_O_3_ cathode is 2171 mW cm^−2^ at 700°C, which is similar to that of the cell using the cathode with *x* = 0.6 (BaZr_0.4_Co_0.6_O_3_ cathode). Further increasing the *x* value to 0.8, the PPD of the cell drops to 1456 mW cm^−2^ at 700°C due to the reduction of BCO/BZCO interfaces. Table 2 shows the BCO and BZCO molar ratio in the BaZr_1−_ *_x_* Co*_x_* O_3_ cathodes and its correlation with the PPD. The more the interface, the higher the PPD. Two phases are formed with the Co‐doping content reaching 40 mol% in BaZrO_3_. For the nominal BaZr_0.6_Co_0.4_O_3_, the molar ratio of BCO and BZCO in the nominal BaZr_0.6_Co_0.4_O_3_ is 14% and 86%. With the formation of the BCO/BZCO interface, the performance of BaZr_0.6_Co_0.4_O_3_ is higher than the pure phase BCO and BZCO. With the increase of the Co‐doping amount in BaZrO_3_, the ratio of BCO decreases, and the percentage of BZCO increases, leading to more BCO/BZCO interfaces. The increased interfaces lead to higher fuel cell performance, suggesting the importance of the interface for the cathode performance. BaZr_0.4_Co_0.6_O_3_, which is consisted of 43 mol% BCO and 57 mol% BZCO, contains the largest amount of interfaces. For BaZr_0.3_Co_0.7_O_3_, that is *x* = 0.7, the material contains 57 mol% BCO and 43% mol% BZCO, in which the number of BCO/BZCO interfaces is similar to that for BaZr_0.4_Co_0.6_O_3_. The similar amount of interfaces leads to similar fuel cell performance. Once the BaZr_0.2_Co_0.8_O_3_ (*x* = 0.8) cathode is used, the composition becomes 71 mol% BCO and 29% mol% BZCO. An evident decline in interface numbers can be expected. As a result, the fuel cell performance drops accordingly. The PPD of the fuel cells is closely related to the interface amounts, further confirming the importance of the BCO/BZCO interface for the electrochemical performance of the BaZr_1−_ *_x_* Co*_x_* O_3_ cathodes.

**TABLE 2:** Correlation between the *x* value in BaZr_1−_ *_x_* Co*_x_* O_3_ and the molar ratio of BCO and BZCO in the nanocomposite cathodes and their peak power densities.

Molecular formula	*x* value	Molar ratio of BCO : BZCO	Peak power density at 700°C
BaZr_0.6_Co_0.4_O_3_	0.4	0.14:0.86	1309 mW cm^−2^
BaZr_0.5_Co_0.5_O_3_	0.5	0.29:0.71	1554 mW cm^−2^
BaZr_0.4_Co_0.6_O_3_	0.6	0.43:0.57	2253 mW cm^−2^
BaZr_0.3_Co_0.7_O_3_	0.7	0.57:0.43	2171 mW cm^−2^
BaZr_0.2_Co_0.8_O_3_	0.8	0.71:0.29	1456 mW cm^−2^

## 3 CONCLUSION

Studying the BCO+BZCO composite with DFT calculations at the atomic level, PLD‐generated nanoscale thin‐film examination and prototype fuel cell tests reveal the significance of the interface. DFT calculations reveal that the BCO/BZCO interfaces reduce the energy barrier in ORR compared to single‐phase BCO or BZCO material, which is advantageous for cathode reactions. The BCO/BZCO multilayer thin film investigations, which are utilized to replicate the reaction activities between single BCO and BZCO particles, suggest that the presence of BCO/BZCO interfaces significantly improves the oxygen exchange, resulting in faster cathode reaction kinetics. Consequently, an H‐SOFC employing the BaZr_0.4_Co_0.6_O_3_ cathode that is the BCO+BZCO composite provides an ultra‐high fuel cell performance of 2253 mW cm^−2^ at 700°C. In addition, the cell operates reliably for over 650 h. The current study not only provides a highly efficient cathode material for H‐SOFCs but also shows the performance enhancement process via the creation of interfaces, providing an intriguing avenue for cathode design.

## 4 EXPERIMENTAL SECTION/METHODS

### 4.1 Materials preparations and characterizations

BaZr_1−_ *_x_* Co*_x_* O_3_ (*x* = 0.3, 0.4, 0.5, and 0.6) oxides were prepared by a sol–gel method using metal nitrates as starting materials. Briefly, stoichiometric amounts of metal nitrates were dissolved in distilled water, and citric acid and ethylene diamine tetra‐acetic acid (EDTA) were added to the solution as the complexing agents. The molar ratio between citric acid : EDTA : metal cations was set to be 1.5:1:1. Both citric acid and EDTA are used as complexing agents for cations to avoid any precipitation. In addition, citric acid was also used as a fuel in the following combustion process. The pH value of the solution was adjusted to around 8 with the ammonium solution. The solution was heated under stirring until it ignited, producing ashes that were fired at 1100°C for 3 h. The phase purity of these as‐prepared powders was examined by using XRD (DX‐2700BH, Haoyuan Instrument). TEM (JEM‐2100F) coupled with EDS was used to observe the morphology and the elemental distribution of the powder. BaZr_1−_ *_x_* Co*_x_* O_3_ bars were prepared by sintering the green bars at 1400°C for 6 h for the ECR measurements. The atmosphere was abruptly changed from air to 50%O_2_, and the relaxation time to reach a new equilibrium of conductivity was recorded.

### 4.2 Thin‐film deposition and characterizations

BZCO thin film and BZCO/BCO multilayer films were deposited by pulsed laser deposition (PLD, nanoPLD instrument, PVD Inc.). The KrF excimer pulsed laser source (COMPEX PRO 110 F) was operated at 10 Hz, with a wavelength of 248 nm. The distance between the target and substrate was kept at 60 mm. BZCO thin film and BCO/BZCO multi‐layer films were deposited by PLD on square, one side polished, (001) oriented SrTiO_3_ substrates (HF‐Kejing). The deposition temperature and oxygen pressure were 600°C and 10^−2^ torr, respectively. The TEM sample was prepared by using a focused ion beam (FIB) instrument (Helios G4 UC, Thermofisher). Bright‐field transmission electron microscopy (BFTEM), EDS, and HRTEM were performed on a JEM‐F200 (JEOL, Japan) microscope.

ECR measurements of all the samples were carried out with a digital meter (Keithley 2450 SourceMeter). Two 1×3 mm parallel strips of Ag collector were pasted onto the films. The separation distance was 1 mm. The tests were carried out in a mixture of dry oxygen and nitrogen, with their flow rates controlled by their respective flowmeters. The temperature was maintained at 600°C, and the oxygen pressure was switched from 0.2 to 0.5 atm.

### 4.3 Fuel cell fabrication and characterizations

Fuel cells were fabricated based on the BaCe_0.7_Zr_0.1_Y_0.2_O_3−_ *_δ_* (BCZY)‐electrolyte half‐cell. BCZY electrolyte powder was also prepared by a sol‐gel method. NiO and BCZY were mixed in a weight ratio of 6 to 4 as the anode powder, and 20 wt% starch was added to the anode. Then, the anode powder was co‐pressed with the electrolyte powder to form green bi‐layers. The green bi‐layers were co‐sintered at 1350°C to densify the electrolyte membrane. The cathode BaZr_1−_ *_x_* Co*_x_* O_3_ cathode slurry was painted on the dense BCZY electrolyte, followed a co‐firing at 900°C for 10 min in a microwave sintering furnace. The use of the microwave sintering method allowed the adherence of the cathode to electrolyte at a relatively low temperature with a short dwelling time, restricting the grain growth of the cathode and mitigating the interfacial reactions. Finally, the complete cell with the structure of NiO+BCZY (anode)/BCZY (electrolyte)/ BaZr_1−_ *_x_* Co_x_O_3_ (cathode) was established. The complete cells were tested with H_2_ as the fuel and static air as the oxidant. An electrochemical workstation (Squidstat Plus, Admiral Instrument) was employed to record the cell performance. The electrochemical impedance spectroscopy (EIS) plots of the cells were characterized under the open circuit voltage condition, and the frequency for the EIS measurement was from 1 MHz to 0.1 Hz. After testing, the morphologies of the fuel cell were observed using scanning electron microscopy (SEM, Phenom XL, Thermo Scientific).

### 4.4 First‐principles calculations

All calculations were carried out using density functional theory (DFT) and the VASP (Vienna ab initio simulation package) program.^[^ ^22^ ^]^ For Hubbard's correction, the *U*_eff_ value was set to 3.32 eV for Co.^[^ ^23^ ^]^ The 2×2×2 supercells were used for bulk optimizations. Energy and force convergence criteria were set to 10^−5^ eV and 0.05 eV Å^−1^, respectively. The cutoff energy was set to 520 eV. A vacuum layer with a thickness of 15 Å was constructed, and a gamma centered 4×4×1 K‐point mesh was used for surface calculations, in which the bottom four layers were fixed, while the top two were completely relaxed. The bulk model was used to construct the BCO/BZCO interface. The Gibbs free energies were calculated by using the total energies of different substances on the surface, corrected with the entropic changes (TΔS) to determine the energy at a specific temperature, and then corrected with the difference in zero point energy ($\Delta E_{\mathrm{ZPE}}$) which is derived from the vibrational frequencies, to obtain the final Gibbs free energies according to the equation: $\Delta G\;=\;\Delta E+\Delta E_{\mathrm{ZPE}}-T\Delta S$.

## CONFLICT OF INTEREST STATEMENT

The authors declare no conflicts of interest.

## Supporting information

Supporting Information

## Data Availability

The data that support the findings of this study are available from the corresponding author upon reasonable request.
