# Supplementary material for: Unveiling the importance of the interface in nanocomposite cathodes for proton‐conducting solid oxide fuel cells
Source: Exploration (Beijing). 2024 Feb 1;4(4):20230082. doi: 10.1002/EXP.20230082 (PMC11335467; doi:10.1002/EXP.20230082)
Supplement: Supplementary file 1 — Supporting Information [file EXP2-4-20230082-s001.docx]

Supporting Information

Unveiling the importance of the interface in nanocomposite cathodes for proton-conducting solid oxide fuel cells

Yanru Yin, Yifan Wang, Nan Yang * and Lei Bi *


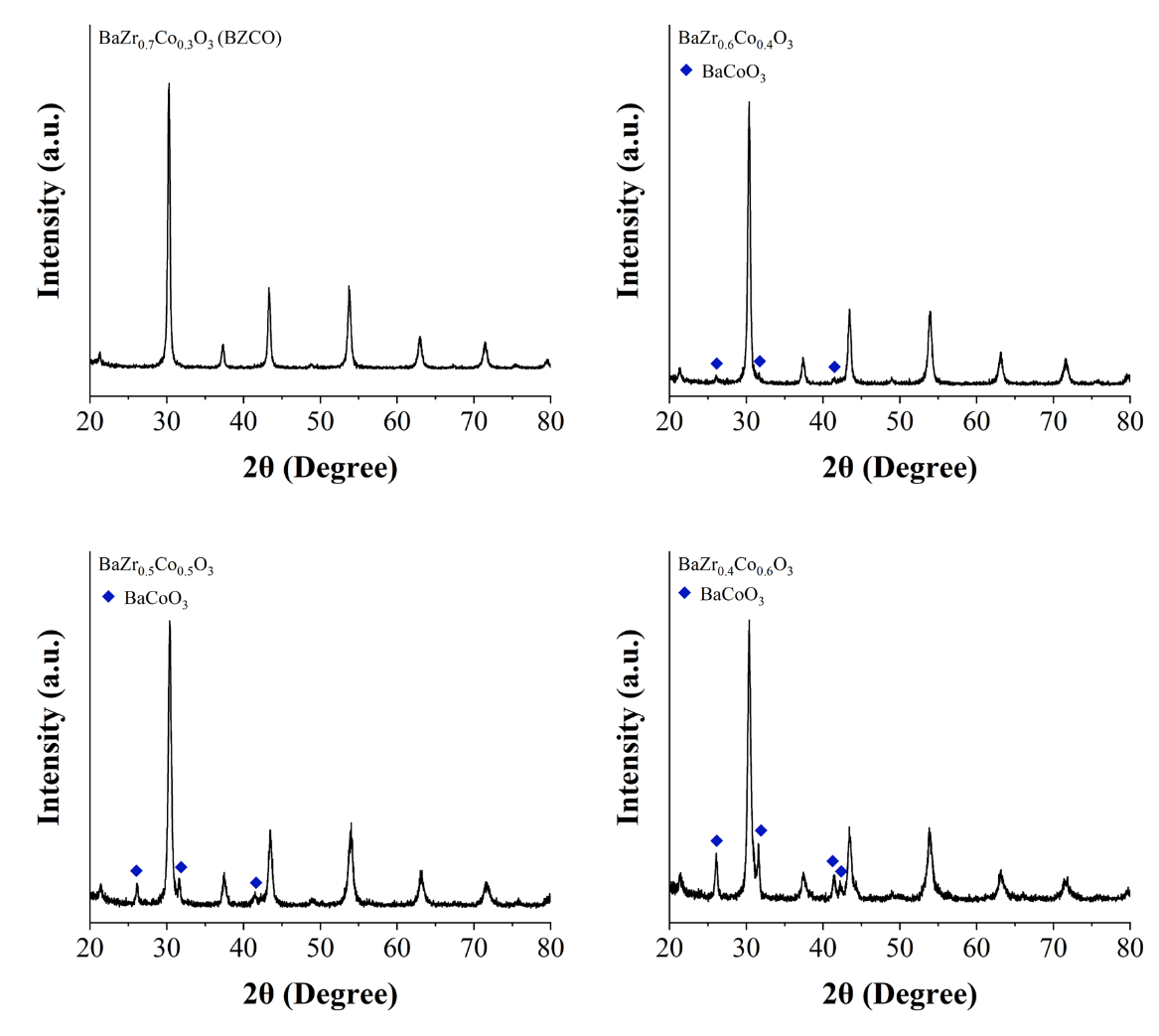


Figure S1. XRD patterns for the as-prepared BaZr_0.7_Co_0.3_O_3_, BaZr_0.6_Co_0.4_O_3_, BaZr_0.5_Co_0.5_O_3_ and BaZr_0.4_Co_0.6_O_3_ powders.


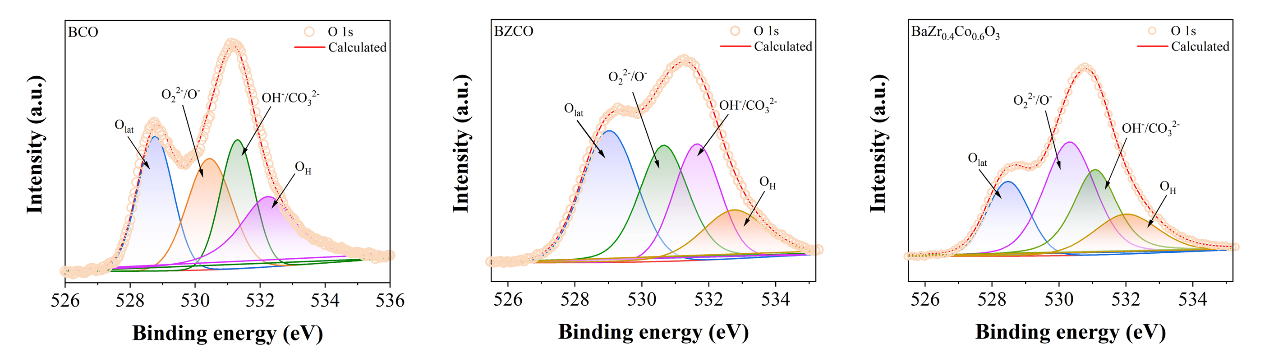


Figure S2. XPS O 1s spectra for BCO, BZCO and BaZr_0.4_Co_0.6_O_3_.


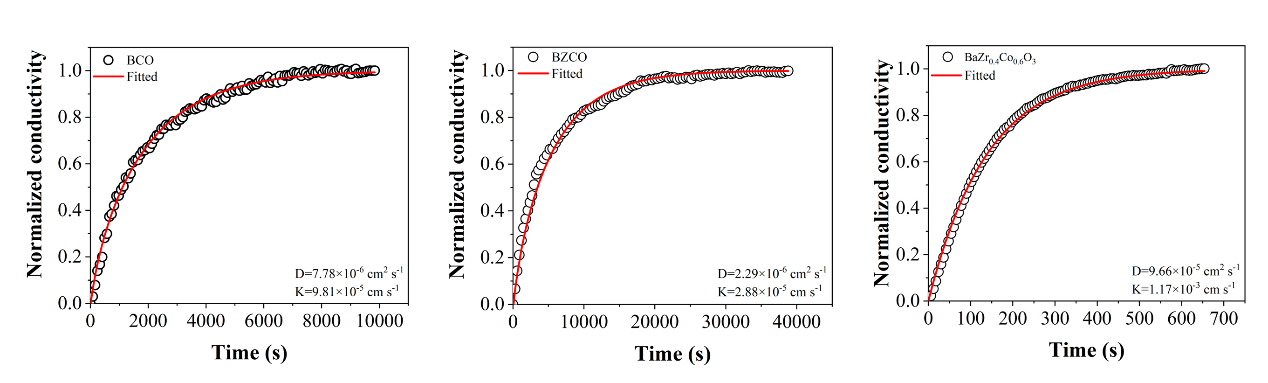


Figure S3. ECR curves for BCO, BZCO and BaZr_0.4_Co_0.6_O_3_ by changing the atmosphere from air to 50%O_2_.

Figure S4. XRD scan of thin film samples.

Figure S5. The conductivity plots of thin films.


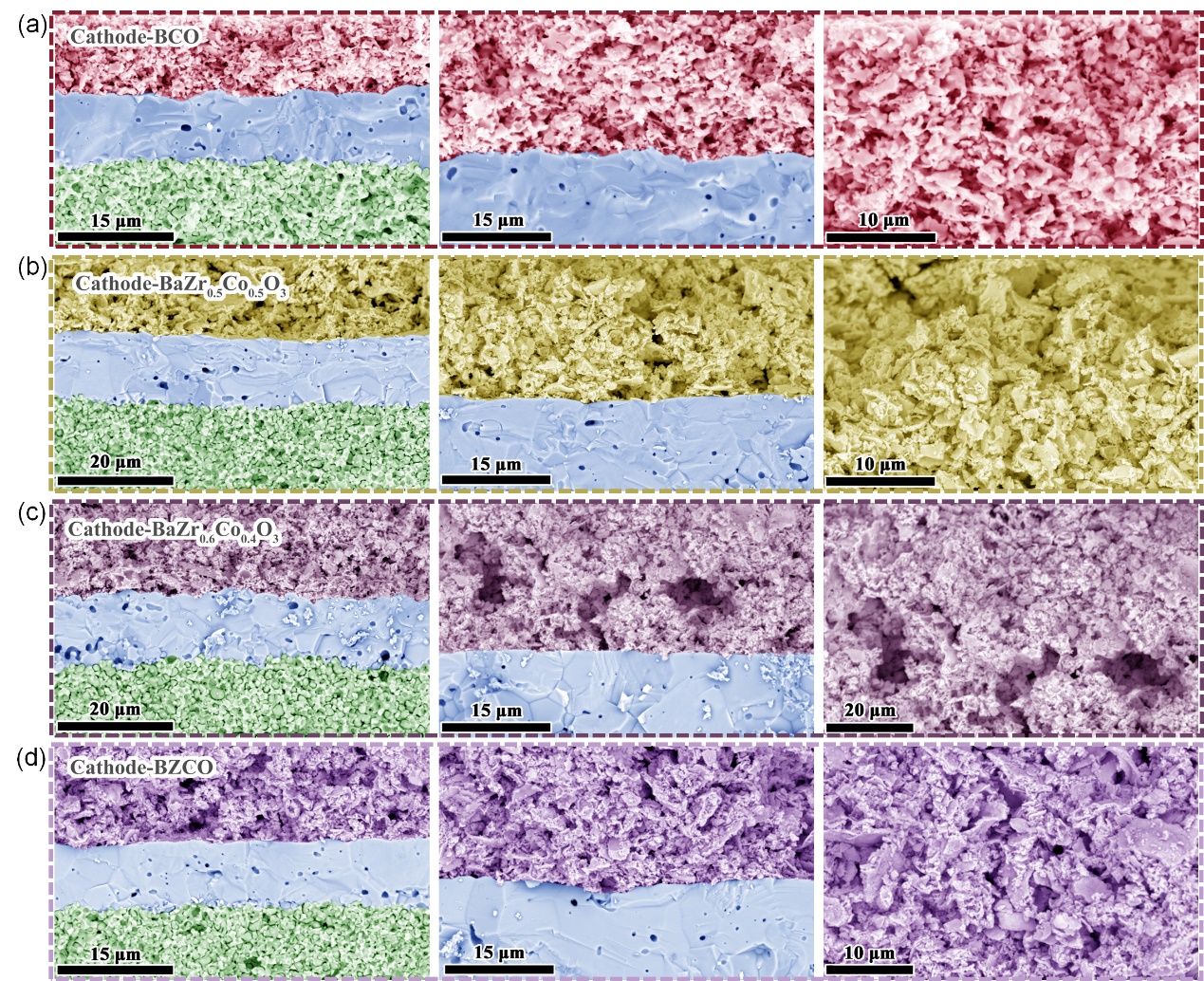


Figure S6. Cross-sectional view for the (a) BCO, (b) BaZr_0.5_Co_0.5_O_3_, (c) BaZr_0.6_Co_0.4_O_3,_ and (d) BZCO cells after testing.


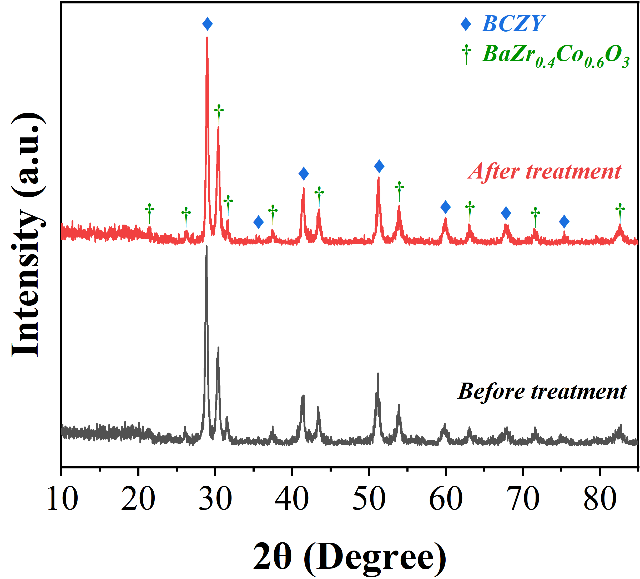


Figure S7. XRD patterns for BaZr_0.4_Co_0.6_O_3_+BCZY composite powder before and after co-firing at 900 ^o^C.


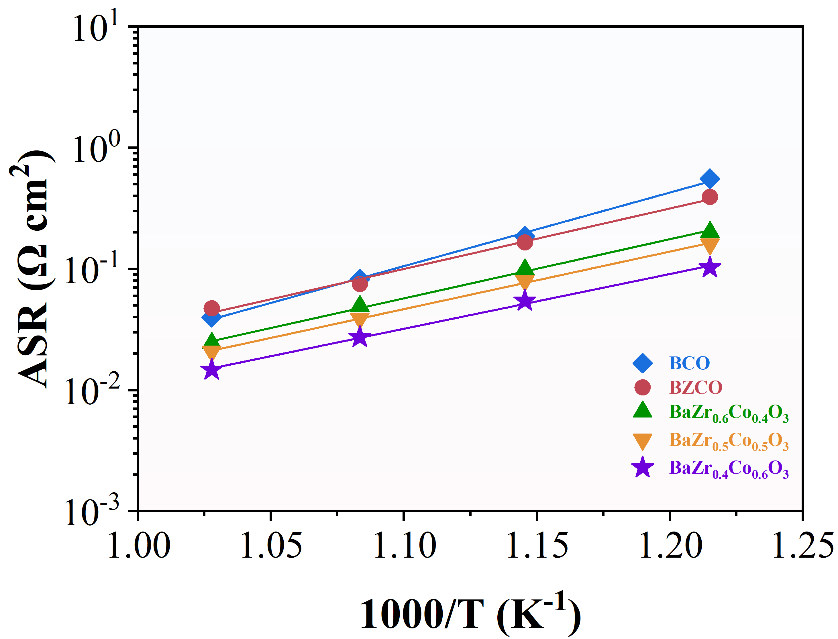


Figure S8. ASR of symmetrical cells using BCO, BZCO, BaZr_0.6_Co_0.4_O_3_, BaZr_0.5_Co_0.5_O_3_ and BaZr_0.4_Co_0.6_O_3_ cathodes tested at different temperatures.


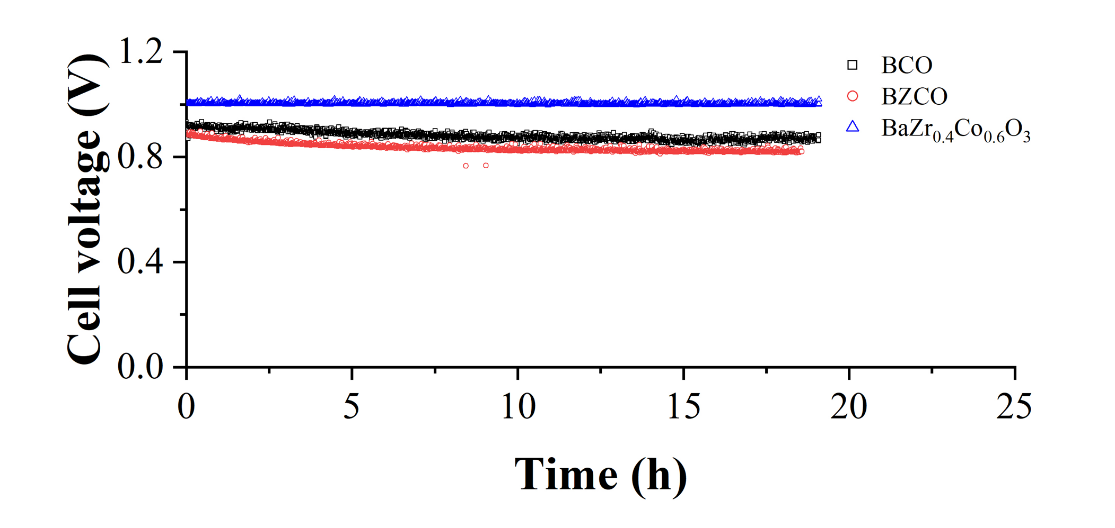


Figure S9. Short-term stability of the cells using different cathodes under the operation condition at 600 ^o^C with an applied current density of 200 mA cm^-2^.

.


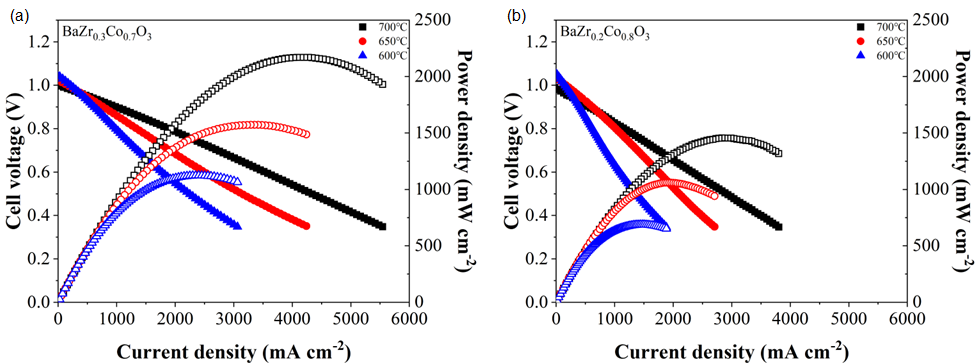


Figure S10. Fuel cell performance of an H-SOFC using (a) BaZr_0.3_Co_0.7_O_3_ and (b) BaZr_0.2_Co_0.8_O_3_ cathodes, respectively.
